# Pyxis Proximity Improves Narcotic Waste Compliance Among Anesthesia Personnel

**DOI:** 10.7759/cureus.66800

**Published:** 2024-08-13

**Authors:** Shruti Parikh, Giacomo Scorsese, Jamie Romeiser, Elliott Bennett-Guerrero, Ana Costa, Morgane Factor

**Affiliations:** 1 Anesthesiology, Renaissance School of Medicine, Stony Brook University, Stony Brook, USA; 2 Anesthesiology, Mount Sinai Health System, New York, USA; 3 Public Health and Preventative Medicine, SUNY Upstate Medical University, Syracuse, USA

**Keywords:** quality assurance, quality improvement, drug diversion, controlled bubstances, narcotic waste

## Abstract

Introduction

Controlled substance waste processes in perioperative areas can be cumbersome. Streamlining this process is a critical component of improving operating room efficiency. At our institution, unused controlled substances require a two-person waste prior to disposal. We hypothesized that access to a centrally located automated medication dispensing system to conduct medication waste would decrease the amount of time required to perform a two-person waste and dispose of unused medications after the completion of a case. We further hypothesized that a decrease in the time required to waste would improve user satisfaction with the waste process.

Methods

This was a single-center, retrospective, IRB-approved quality assurance analysis. Research Electronic Data Capture (REDCap, Stony Brook, NY, United States) software was used to design an anonymous survey, which was distributed via email from 06/09/2021 to 09/09/2021. Wilcoxon signed-rank tests were used to compare before and after paired responses for individuals. Analyses were performed using SAS 9.4 © (Cary, NC, United States) software.

Results

Participants reported a significant decrease in time carrying used narcotics after a surgical case. For the primary outcome, median (IQR) scores demonstrated a significant decrease from 2, representing “10-20 minutes” (IQR 1-3), to 1, indicating “<10 minutes” (IQR 1-2) spent carrying used narcotics (p < 0.0001). Reported satisfaction with the process significantly increased from 2 to 3 points, on a scale of 5, after using the new centrally located Pyxis (p < 0.0001). Participants reported the wasting process as less time-consuming, carried fewer used controlled substances, and were less likely to wait until the end of the shift to waste.

Conclusions

By improving the ergonomics of the waste process after adding a Pyxis system in a more central location, the time anesthesia clinicians spent carrying used controlled substances decreased. Additionally, provider satisfaction increased, likely related to the ease of finding a partner to witness wasted medications.

## Introduction

Narcotics and controlled substances are essential to any anesthesiology practice. The Drug Enforcement Administration is the federal entity charged with enforcing and executing the Controlled Substance Act (CSA) of 1970. The CSA defines five controlled substances schedules, or classes, based on a substance’s medical use, the potential for abuse, and safety or dependence liability [1]. Healthcare facilities that use these medications in a clinical setting require a regulated process to waste excess medication.

In general, 10-14% of American healthcare professionals have some form of substance use disorder, and intravenous opioids are the most common drug of abuse [2]. Operating suites, ambulatory procedure sites, and surgical centers are places where opioids and other controlled substances are frequently used for routine anesthetics. Inefficient wasting processes for controlled substances may lead to medical errors and patient safety issues. It could also inadvertently facilitate diversion, worsening the nationwide substance abuse epidemic. Specifically, a failure modes and effects analysis for potential sources of controlled substance diversion yielded 2,013 potential failure modes, including a high risk of potential diversion for a witness waste process [3].

There is an unmet need to simplify the waste process. The geographical location of the automated medication dispensing system, where narcotics are wasted, was identified as an initial barrier to the waste process at our institution. As a result, a new dispensing system was introduced to the post-anesthesia care unit (PACU). Our study had several objectives: first, we hypothesized that the introduction of the new dispensing system would decrease the amount of time clinicians carry opioids requiring disposal. We also hypothesized that there would be a differential change between provider groups in the amount of time they carry opioids that require disposal, specifically that anesthesiology residents would perceive the most beneficial change from the reduction of time carrying opioids compared to attendings and certified registered nurse anesthetists (CRNA). Lastly, we hypothesized that access to this additional, centrally located system would positively impact clinician satisfaction and reduce perceived logistical obstacles to the controlled substance waste process.

## Materials and methods

Study approval, setting, and participants

This was a single-center, retrospective quality assurance analysis. It was approved by the Stony Brook University Hospital Quality Assurance/Quality Improvement Committee and Institutional Review Board (IRB2022-00122). Stony Brook University Hospital (SBUH) is a 788-bed facility in Long Island, NY, United States. The hospital has 25 main ORs and numerous off-site procedural suites and anesthetizing locations, including endoscopy, interventional radiology, ambulatory surgery, and labor and delivery. The hospital performs roughly 36,000 procedures a year across a wide breadth of surgical sub-specialties. Our study examined the controlled substance waste practices in our main ORs and PACU.

Survey development and distribution

Research Electronic Data Capture (REDCap, Stony Brook, NY, United States) software was used to design an anonymous survey. REDCap is a secure, self-service web application that enables users to build and perform data collection for projects and surveys. The application is licensed to SBUH under a consortium agreement with Vanderbilt University. Our team utilized the REDCap software to develop a 20-question survey on user practices and user satisfaction pre- and post-intervention (Appendix A).

The survey was distributed to all main OR and PACU anesthesia clinicians who can perform controlled substance wasting using the PyxisTM (© BD, Franklin Lakes, NJ, United States), an automated medication dispensing system used at our institution. This includes attending anesthesiologists, anesthesiology residents, and CRNAs. The surveys were distributed via email and were accessible via a smartphone, tablet, or computer. Data collection was performed over three months. Survey questions assessed several issues associated with the previous controlled substance-wasting process at a remotely located Pyxis machine. Participants were asked to compare their experiences before and after adding a more centrally located Pyxis for controlled substance waste in the PACU. The surveys evaluated were limited to those who answered both the pre- and post-intervention components.

Primary outcome

The primary outcome was a change in the time participants spent carrying disposable controlled substances. This was assessed by asking participants to compare how long, on average, they carried medications that require wasting after the end of a case before and after introducing a PACU Pyxis system with a controlled substance waste bin.

Secondary outcomes

The secondary endpoints examined changes in provider satisfaction and perceived benefits reported by the implementation of the PACU Pyxis System. Questions assessed changes in perception of how time-consuming the process is, change in how long it takes to find a partner to witness the wasting of used controlled substances, change in how often end-of-shift wasting occurs, and change in the maximum number of medications carried at once.

Statistical analysis

The demographics of survey participants are described as frequencies and percentages. Wasting partner practices were summarized by anesthesia clinician groups. Medians and IQRs were computed for the 6 Likert scale survey questions. Wilcoxon signed-rank tests were used to compare before and after paired responses for individuals at a significance level of 0.05. Paired responses for the primary outcome (time carrying used substances) were assessed in several subgroup analyses using Wilcoxon signed-rank tests. Change in time carrying used substances was also compared across the three provider groups using the Kruskal-Wallis test, and separate group-by-group comparisons were conducted using Wilcoxon rank sum tests. These analyses were conducted using a reduced alpha of 0.017 to adjust for multiple comparisons. Analyses were performed by a statistician (co-author, JR) using SAS 9.4 © (Cary, NC, United States) software.

## Results

A total of 148 surveys were submitted. Respondents identified as OR nurses (n = 18) and PACU nurses (n = 20) were excluded from this analysis to target the investigation toward anesthesia clinicians only. Fourteen attending anesthesiologists reported that they did not waste controlled substances and were excluded from the analysis. An additional 18 participants did not fully complete the post-intervention survey and were also excluded. The differences in the response distribution for the pre-intervention survey were negligible after excluding the incomplete surveys. The final number of surveys for analysis was 78. Participants represented a mix of attending anesthesiologists, anesthesiology resident physicians, and CRNAs. Forty-eight participants (62%) reported wasting controlled substances using the new PACU Pyxis controlled substance waste bin (Table 1).

**Table 1:** Demographic breakdown of survey respondents CA-1: clinical anesthesia resident year 1; CA-2: clinical anesthesia resident year 2; CA-3: clinical anesthesia resident year 3; CRNA: certified registered nurse anesthetist; PACU: post-anesthesia care unit

	n	%
Total surveys	78	
Role		
Attending	20	26%
Resident	33	42%
CRNA	25	32%
Years		
Attending		
1-4 years	5	25%
5-9 years	4	20%
10+ years	11	55%
Resident		
CA-1	15	45%
CA-2	8	24%
CA-3	8	24%
CRNA		
1-4 years	9	36%
5-9 years	3	12%
10+ years	13	52%
Time to waste narcotics (min)		
1-5	21	27%
6-10	12	15%
11-20	10	13%
Have you wasted narcotics in the new PACU Pyxis?		
Yes	48	62%
No	29	37%

A total of 91% of attending anesthesiologists, 60% of CRNAs, and 60% of residents reported “never” wasting controlled substances with a registered nurse (RN). Additionally, 20% of attending anesthesiologists, 16% of CRNA, and 9% of residents reported that they “rarely” waste with an RN. Our survey revealed that CRNAs most often wasted with other CRNAs and anesthesiology residents most often wasted with other anesthesiology residents (Table 2).

**Table 2:** Demographic breakdown of waste partner CRNA: certified registered nurse anesthetist

For a two-person waste, how often is the second person a nurse?	n	%	Attending (n = 20)	Resident (n = 33)	CRNA (n = 25)
Never	57	73%	60%	91%	60%
Rarely	11	14%	20%	9%	16%
Sometimes	5	6%	15%	0%	8%
Most of the time	5	6%	5%	0%	16%
Always	0	0%	0%	0%	0%
For a two-person waste, how often is the second person a CRNA?	n	%	Attending (n = 20)	Resident (n = 33)	CRNA (n = 25)
Never	7	9%	0%	21%	0%
Rarely	27	35%	15%	70%	4%
Sometimes	23	29%	65%	9%	28%
Most of the time	19	24%	20%	0%	60%
Always	2	3%	0%	0%	8%
For a two-person waste, how often is the second person a resident?	n	%	Attending (n = 20)	Resident (n = 33)	CRNA (n = 25)
Never	5	6%	0%	0%	0%
Rarely	8	10%	0%	0%	32%
Sometimes	24	31%	60%	0%	48%
Most of the time	31	40%	40%	70%	0%
Always	10	13%	0%	30%	0%

**Figure 1:**
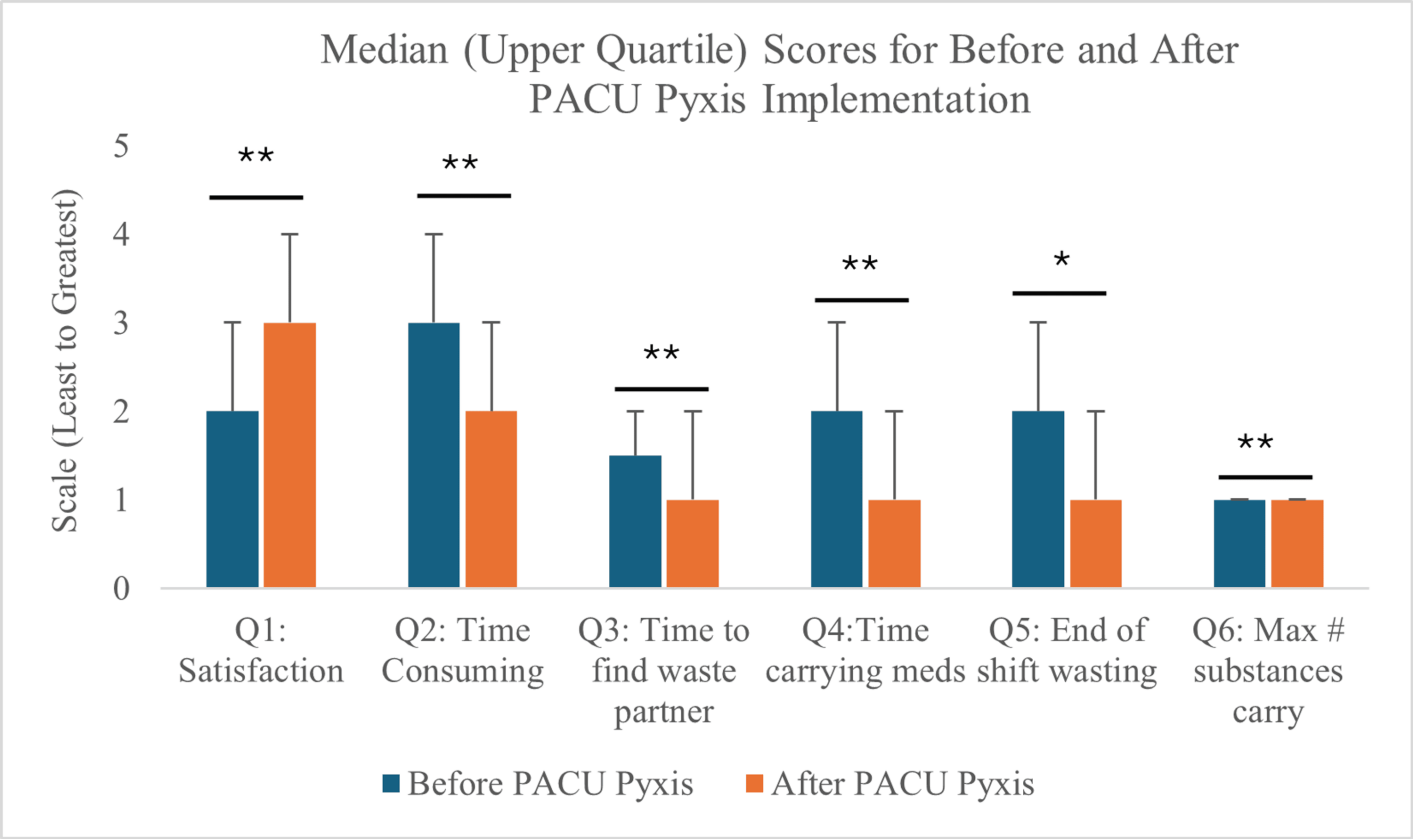
Median score for before and after questions Survey questions with before and after IQR. Q1: clinician satisfaction before 2 (IQR 2-3) and after 3 (IQR 2-3). Q2: perceived time consumption of waste process before 3 (IQR 2-4) and after 2 (IQR 2-3). Q3: time needed to find a waste partner before 1.5 (IQR 1-2) and after 1 (IQR 1-2). Q4: time spent carrying used narcotics before 2 (IQR 1-3) and after 1 (IQR 1-2). Q5: how often did the clinician wait till the end of the shift to waste before 2 (IQR 1-3) and after 1 (IQR 1-2)? Q6: the maximum number of controlled substances carried before 1 (IQR 1-1) and after 1 (IQR 0-1). Wilcoxon signed rank significance testing ** p < 0.0001; * p = 0.0002 PACU: post-anesthesia care unit

After the implementation of a centralized Pyxis in our PACU with an adjacent controlled substance waste bin, participants reported carrying used controlled substances for shorter periods after the end of a case. Median (IQR) scores demonstrated a significant decrease from 2, representing “10-20 minutes” (median IQR 1-3]) to 1, indicating “<10 minutes” (median IQR 1-2) spent carrying used controlled substances (p < 0.0001). There was a significant increase in provider satisfaction with the controlled substance waste process. Median (IQR) survey scores increased significantly from 2 (median IQR 2-3) “Somewhat dissatisfied” to 3 (median IQR 2-3) “Neutral” when asked to describe their satisfaction with the wasting process (p < 0.0001). Participants felt less burdened by the wasting process, moving from a median (IQR) score of 3 (median IQR 2-4), representing the process as “Time-consuming,” to a score of 2 (median IQR 2-3) representing the process as “Somewhat time-consuming” (p < 0.0001). There was a reduction in time to find a waste partner from five to 15 minutes down to five minutes (median IQR change 1.5 to 1 on the Likert scale, p < 0.0001). After the implementation of the new Pyxis, there was a lower tendency to wait until the end of the shift to waste (p = 0.0002), and on average, participants carried fewer controlled substances throughout the day (p < 0.0001) (Figure 1).

We further examined these six questions with those who reported using the new Pyxis (62% of respondents) and those who did not (37% of respondents). Among the subgroups of users, all results remained consistent and significant. Among the subgroup of non-users, the only benefit that remained was a reduction in the carrying time of used controlled substances.

The carrying time of used controlled substances was also examined within the three clinician groups. Subgroup analyses demonstrated that both anesthesiology residents and CRNAs experienced a significant reduction of used controlled substance carrying time. For anesthesiology residents, the median change was -1 (median IQR change -1, 0) and p < 0.0001. For CRNAs, the median change was 0 (median IQR change -1, 0) and p = 0.001. However, the change in used controlled substance carrying time was not significant among attendings with a median change of 0 (median IQR change -0.5, 0) and p = 0.06. There was no significant difference in the change in carrying time across the three groups (Kruskal-Wallis p value = 0.08). Introducing a new PACU Pyxis seemed to have a greater impact on residents’ carrying time than attendings (Wilcoxon rank sum p = 0.03), but this difference did not reach the prespecified alpha of p = 0.017.

## Discussion

In our single-center, IRB-approved, retrospective quality assurance analysis, we sought to investigate potential benefits to the wasting process after implementing a new Pyxis system in the PACU. Placing a new Pyxis in a more central location decreased the time spent carrying narcotics after the end of a case, with an increase in user satisfaction with the wasting process. Those who used the new Pyxis found the waste process to be less time-consuming and spent less time finding a second person to witness the waste.

Anesthesiologists have historically been disproportionately affected by substance abuse, despite hours of formal education and strict control measures [4,5]. Moreover, substance abuse is a potentially lethal occupational hazard for anesthesiology residents. Approximately 80% of United States anesthesiology residency programs report some experience with impaired residents, and 19% report at least one pretreatment fatality due to illicit drug addiction [6]. Therefore, workflow strategies that discourage the diversion of controlled substances should be prioritized. However, guidance regarding the disposal of controlled substances has generally lacked a unified policy. Institutions must individually navigate the numerous regulations to assure compliance and build practical and appropriate procedures into their workflow [7].

A 2017 conference on drug diversion by anesthesiologists by the Anesthesia Patient Safety Foundation identified several risk management strategies designed to reduce the potential for diversion. One such approach suggested that anesthesia clinicians should carefully consider whether to keep controlled substances with high diversion potential on one’s person after they have been used [8]. This highlights the need for a waste process system that facilitates fast and efficient disposal to reduce the amount of time one needs to carry controlled substances after the conclusion of a case. In addition, an efficient waste process in any operating suite or surgical center would help reduce medical errors and discourage the diversion of controlled substances.

Controlled substance diversion can attract high-profile negative attention to a healthcare system, be emotionally and financially expensive for individuals involved, and ultimately be detrimental to patient care. In a severe instance, acts of diversion by a single provider resulted in five cases of bacteremia with an identical pathogen traced back to tampered fentanyl and hydromorphone syringes. Of the patients affected, one expired due to complications of sepsis [9]. Recent advancements in automated medication dispensing systems include incorporating data analysis to identify and flag anomalous activity associated with narcotic diversion [10]. Machine learning has also been implemented to create algorithms that identify patterns of narcotic activity that are at high risk of diversion [11].

In general, the ability to dispose of controlled substances quickly and safely will improve efficiency and potentially reduce the diversion of controlled substances. In addition, we attempted to identify the barriers that remained after implementing the new Pyxis in the PACU. The added time of finding a partner to waste with can interrupt the typical clinical workflow, particularly when the institution is experiencing a surge in patient volume. This becomes particularly stressful for anesthesia personnel who are turning over an OR in between cases with limited time to search for a second person to waste with. We not only found that provider satisfaction (secondary outcome) increased with improving the ergonomics of the waste process by adding a Pyxis system in a central location, but the time anesthesia providers spent carrying used controlled substances decreased significantly (primary outcome). This decrease was likely related to the ease of finding a partner to witness the waste process. The likelihood of finding someone to waste with is higher in a central location such as the PACU, where there is a high volume of anesthesia personnel, compared to the isolated location of the original Pyxis. From a compliance standpoint, it is preferred that anesthesia clinicians not use controlled substances for prolonged periods after a case is completed. It is recommended that they dispose of these medications as soon as possible once they finish providing anesthesia care. By promptly discarding used narcotics, there is a decreased chance of losing the syringes or performing medication errors.

Another area for potential improvement is to revisit the need for a second person to witness the waste. While flexibility regarding the implementation of the controlled substance waste process lies with the hospital, the Code of Federal Regulations, Title 21, Chapter 2, Part 1317, dictates that the destruction of controlled substances must be witnessed and documented by at least two employees and that the controlled substances are rendered non-retrievable [12]. However, in our institution, a significant barrier to the destruction of controlled substances was the availability of a second witness. The prior waste location was located in the sterile environment of the main OR, which was inaccessible by PACU nurses. We found that after the implementation of a centralized Pyxis in our PACU, there was a significant increase in clinician satisfaction with the waste process. While not the focus of this study, it is interesting to note that Raman spectroscopy could potentially allow for rapid, inexpensive, and nondestructive analysis of controlled substances by a single clinician [13]. Such an anti-diversion tool could make the two-person waste process obsolete by using laser technology to analyze the chemical fingerprint of a medication [14]. The potential for a one-person waste process has the potential to significantly improve the waste process and improve OR efficiency.

Based on our literature review, no studies have examined clinician satisfaction in the controlled substance waste process, which is a task that has the potential to cause delays and inefficiencies if found to be too time-consuming. A strength of our analysis was that in surveying a wide array of OR anesthesia personnel, we identified resident physicians as a specific subgroup dissatisfied with the wasting process, as they work the most overnight and weekend shifts, which are times when it is the most difficult to find a second person as a witness. The ability to waste in PACU alleviates this burden since there will always be PACU nurses available to waste with at the conclusion of a case, regardless of the time of the day. Another strength of our study was the timing of the survey deployment. We deployed the survey approximately three months after introducing the new Pyxis. This allowed time for individuals to use the new system while also minimizing the recall period. We also assessed responses from those who used the new Pyxis and those who had not yet had the opportunity. Not surprisingly, there was a clear perceived benefit among those who had used the new system but not among those who had not. These findings provide internal validity to our study.

Our study was limited to only surveying our home institution. Furthermore, we could only survey those who work in the main OR. Off-site areas such as endoscopy, interventional radiology, special procedures, ambulatory surgery, and labor and delivery were not included due to the different waste processes used at these sites [15].

Due to the nature of our specialty, the process of wasting controlled substances is a necessary part of practicing anesthesiology. By identifying limitations to the timely wasting of these medications, we hope to better streamline this process and improve OR efficiency and patient safety. Adding a Pyxis in the PACU at our institution decreased the time required to waste medication and increased user satisfaction.

## Conclusions

The addition of a new Pyxis in the PACU led to timely and more compliant wasting of narcotics after the completion of a case. This also resulted in improved user satisfaction among anesthesia personnel who use and waste these medications as a part of their daily practice. The benefit of Pyxis proximity was further highlighted among residents who work overnight call shifts, during which there is limited time to walk to a remote Pyxis and fewer personnel available to witness a waste with.

Improving the narcotic waste process has the potential to increase operating room efficiency and improve patient safety. Moving the Pyxis to a central, high-traffic area is a simple and low-cost intervention that is applicable to every institution where narcotics are used by anesthesiologists in the perioperative setting.

## Figures and Tables

**Figure 2:**
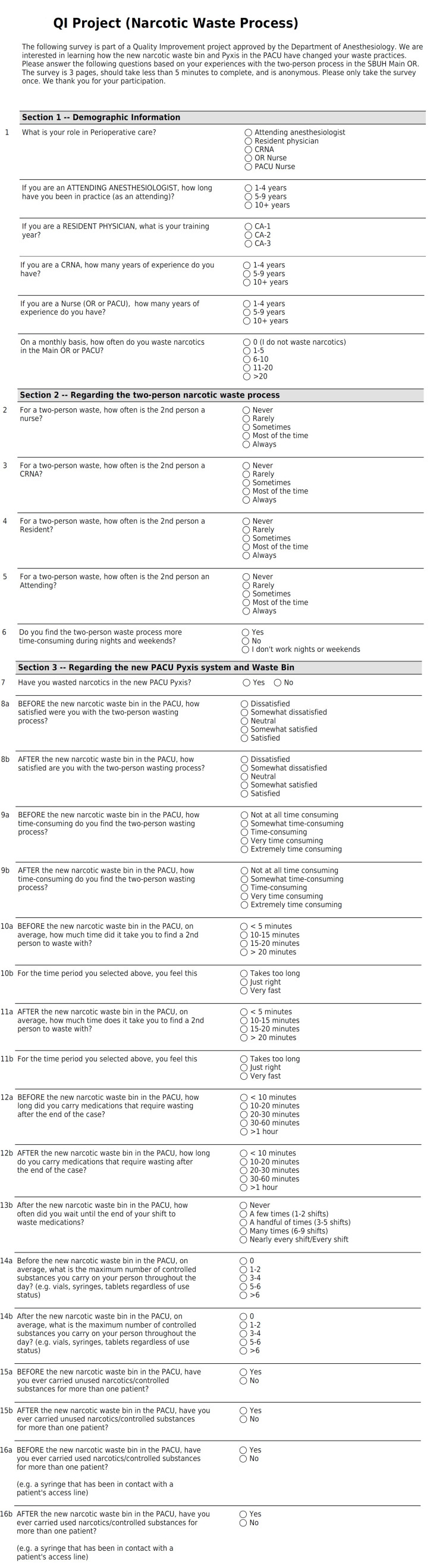
Copy of the REDCap survey CA-1: clinical anesthesia resident year 1; CA-2: clinical anesthesia resident year 2; CA-3: clinical anesthesia resident year 3; CRNA: certified registered nurse anesthetist; PACU: post-anesthesia care unit; REDCap: Research Electronic Data Capture

## Appendices

Appendix A
